# Moderately enhancing cytokinin level by down-regulation of *GhCKX* expression in cotton concurrently increases fiber and seed yield

**DOI:** 10.1007/s11032-015-0232-6

**Published:** 2015-01-29

**Authors:** Juan Zhao, Wenqin Bai, Qiwei Zeng, Shuiqing Song, Mi Zhang, Xianbi Li, Lei Hou, Yuehua Xiao, Ming Luo, Demou Li, Xiaoying Luo, Yan Pei

**Affiliations:** Biotechnology Research Center, Southwest University, No. 2 Tiansheng Road, Beibei, Chongqing, 400716 People’s Republic of China

**Keywords:** CKX, Cytokinin, Cotton fiber, Seed, Yield

## Abstract

Cotton is the leading natural fiber crop in the world. Cotton seeds are also an important oil and protein source. However, enhancement of fiber abundance usually leads to a smaller seed. Thus, it has become a challenge for cotton breeding to concurrently increase fiber yield and seed yield. To improve cotton yield, we elevated the endogenous cytokinin level in transgenic cotton by constitutive suppression of cytokinin dehydrogenase (CKX), a key negative regulator controlling endogenous cytokinin in plants. The slightly and moderately suppressed transgenic cotton plants showed normal growth and development, while the severely suppressed plants exhibited a typical cytokinin-overproduction alteration. The suppression of CKX led to an enhancement of endogenous cytokinins in transgenic cotton plants. Total cytokinins in moderately suppressed lines, CR-3 and CR-6, increased by 20.4 and 55.5 % respectively, and that in the severely suppressed line (CR-13) increased by 134.2 % compared to the wild type. The moderately suppressed lines showed a delay in leaf senescence, higher photosynthesis, more fruiting branches and bolls, and bigger seed size. Field trials showed that seed yield and lint yield of the moderately suppressed CR-6 line increased by 15.4 and 20.0 %, respectively. Meanwhile, the enhanced cytokinin level in transgenic cottons did not show significant influence on fiber qualities. Our data demonstrated that *CKX* is a promising gene for crop yield improvement.

**Electronic supplementary material:**

The online version of this article (doi:10.1007/s11032-015-0232-6) contains supplementary material, which is available to authorized users.

## Introduction

Cotton is one of the most valuable commercial crops in the world. In addition to the fiber used for textile manufacturing, cotton seed is an important source of oil and protein, and the seed hulls are used for cattle feed and mushroom production (Sunilkumar et al. 2006; Wilkins et al. 2000). Cotton fiber is derived from single epidermal cells of seeds. Enhancing the abundance of fiber usually leads to smaller seeds (Miller and Rawlings 1967; Zhang et al. 2005). Thus, it is a challenge for cotton breeding to concurrently increase both fiber yield and seed yield.

Cytokinins are a group of phytohormones that regulate cell division and influence numerous developmental and physiological processes of plants (Werner and Schmülling 2009), including leaf senescence (Gan and Amasino 1995), vascular development (Mähönen et al. 2006), cell differentiation at shoot and root apical meristem (Wolters and Jürgens 2009), nutrient uptake and allocation (Séguéla et al. 2008), abiotic (Peleg et al. 2011; Rivero et al. 2007) and biotic stress responses (Choi et al. 2010; Siemens et al. 2006), and regulation of source–sink relationships (Roitsch and Ehneß 2000). Importantly, recent studies have revealed that cytokinin is a key regulator for seed yield (Ashikari et al. 2005; Bartrina et al. 2011).

Exogenous application of kinetin (6-furfurylaminopurine), one of the cytokinin compounds, was able to improve the yield of seed cotton and lint fiber (Sawan et al. 2000). However, large-scale commercial applications of cytokinins in crops are unfeasible in practice due to high costs and time consumption (Li et al. 2004). Genetic modification provides a means to manipulate hormone concentrations in plants. Promoting cytokinin biosynthesis is an effective method of overproducing cytokinin in plants. The *ipt* gene encoding isopentenyl transferase, which catalyses the rate-limiting step in cytokinin biosynthesis, has been widely used for the enhancement of cytokinins in transgenic plants (Klee et al. 1987; Smigocki and Owens 1988; Smigocki 1991; Wang et al.^1997^; Geng et al. 2001, 2002; van der Graaff et al. 2001; Kuppu et al. 2013; Reguera et al. 2013; Rupp et al. 1999; Synková et al. 1999). However, constitutive overexpression of the gene is usually associated with adverse effects on growth and development of the plants (Smart et al. 1991; Medford et al. 1989; McKenzie et al. 1994; van der Graaff et al. 2001; Geng et al. 2001; Guo et al. 2005; Synková et al. 1999). Another approach to elevating endogenous cytokinins is the suppression of cytokinin deactivation. Cytokinin oxidase/dehydrogenase (CKX), which catalyzes the catabolism of cytokinins to inactive products that lack the N^6^-unsaturated side chain (Jones and Schreiber 1997), is a crucial negative regulator controlling endogenous cytokinin contents in the plant kingdom (Mok and Mok 2001; Schmülling et al. 2003; Werner et al. 2001, 2003; Kowalska et al. 2010). Decrease of *CKX* expression level results in more cytokinins in inflorescence meristems, and thus more grains (Ashikari et al. 2005).

To increase endogenous cytokinins in cotton, we had tried to generate transgenic cottons using a constitutive promoter, CaMV35S, to control the expression of *ipt*. However, as happened in cucumber transformed with *35S::ipt* reported by Smigocki and Owens (1989), the transformed calli never developed further into plant regenerants (unpublished data). In this paper, by using *GhCKXRNAi*, we successfully generated cytokinin-overproduction cottons. Our data demonstrated that moderate suppression of *GhCKX* can significantly improve both seed and fiber yield of cotton.

## Materials and methods

### Plant transformation and growth conditions

The *35S::GhCKXRNAi* construct (Zeng et al. 2012) was introduced into cotton using the method of *Agrobacterium*-mediated transformation as described previously (Luo et al. 2007). Kanamycin-resistant and GUS-positive plants were screened out for further study. Cotton plants were grown in greenhouse in 30 × 28 cm (diameter × height) pots under a 16-h light/8-h dark cycle, at 26–32 °C. Pindstrup Substrate (Pindstrup Mosebrug A/S, Denmark) was used as potting mixture. The plants were watered once every 2 days, and fed once every month with compound fertilizer (XIYANG 17-17-17, China).

### RNA isolation and RT-PCR analyses

Total RNA was isolated from stem shoots according to the manual of EASY Spin Plant RNA Kit (Aidlab, China). cDNA was synthesized by RevertAid First Strand cDNA Synthesis Kit (Fermentas, Canada). For RT-PCR analysis, the cDNA sample was used to amplify a *GhCKX* segment with primers GhCKX1 (5′-ccaaagggcacggtcattctg-3′) and GhCKX2 (5′-cccctttcggtgctggttg-3′). The *HISTONE3* gene (AF024716) was used as the internal control with primers GhHIS1 (5′-gaagcctcatcgataccgtc-3′) and GhHIS2 (5′-ctaccactaccatcatggc-3′). Each reaction contained 10 µL SsoFast EvaGreen Supermix (Bio-Rad, USA), 500 nM each of forward and reverse primers and 1 μL of cDNA. PCR was carried out on a CFX96™ Real-Time System with following conditions: a pretreatment (95 °C, 2 min) followed by 40 amplification cycles (95 °C, 15 s; 56 °C, 15 s; 72 °C, 20 s).

### Quantification of endogenous cytokinins

The concentration of endogenous cytokinins was determined in young leaves (the first main-stem leaf from the apex at 130 days after sowing [DAS]), mature leaves (the fifth main-stem leaf from the apex at 130 DAS), stem shoots (the first internode from the apex at 90 DAS), flower buds (3 days before anthesis) and 35-DPA (days post-anthesis) ovules. Tissues (100 mg) were pooled for each sample. Three independent biological replicates were analyzed for the tissue of each line. Extraction, purification, and quantification of endogenous cytokinins by high-performance liquid chromatography linked to a 4000 Q TRAP LC/MS/MS system (ABsciex, USA) were performed according to the method described by Zeng et al. (2012). The product ion pairs of each cytokinin component and corresponding deuterated internal standards, as well as their acquisition parameters, are summarized in Table S4.

### Determination of chlorophyll contents

Leaves (100 mg fresh weight (FW)] were placed in a 10 mL tube containing 5 mL 80 % acetone and incubated in the dark at room temperature until the tissues became white. Total chlorophyll was determined using absorbance at 645 nm and 663 nm according to the equation: 20.2 A_645_ + 8.02 A_663_ (Chory et al. 1994).

### Photosynthesis measurements

Photosynthetic rate was measured by the Li-6400 portable photosynthesis system (LI-COR, Lincoln, NE, USA). The fourth main-stem leaf from the apex (functional leaf) of 10 plants per line at 120 DAS was used for the analysis. The instrument was set at saturating light of 1,200 μmol m^−2^ s^−1^ and a CO_2_ concentration of 400 ppm.

### Determination of soluble sugar and soluble protein

Ovules and boll shells of 35-DPA bolls at third fruit branch (base to top) were frozen in liquid nitrogen. Samples (100 mg FW of each) were homogenized and extracted in 5 mL 80 % ethanol, and heated for 45 min in boiling water. After cooling to room temperature, the extraction was centrifuged at 3,000 rpm for 10 min. The supernatant was collected to determine the amount of soluble sugars by the colorimetric method with anthrone-sulfuric acid at 620 nm with glucose as a standard.

The protein content was determined by the Coomassie Brilliant Blue G-250 method with bovine serum albumin as the standard. Soluble proteins were extracted from 0.2 g of fresh tissue at 4 °C with 5 mL of extracting solution containing 93.7 % 0.2 M Na_2_HPO_4_, 5.2 % 0.2 M NaH_2_PO_4_, 2 % PVP, and 0.1 % (v/v) β-mercaptoethanol for 60 min. After centrifugation at 4,000 rpm for 10 min, the supernatant was used for protein determination.

### Ovule culture

Ovules at 0 DPA were harvested and cultured in BT medium according to the method of Beasley and Ting (1974). As well as 0.5 μM gibberellic acid and 5 μM indole-3-acetic acid, trans-Zeatin with concentrations of 0, 0.5, 1.0, 5.0, 8.0, and 15 μM was added to the medium. After 2-week culture in the dark at 32 °C, ovules were observed by an MVX-10 microscope (Olympus, Japan).

### Scanning electron microscopy (SEM)

The 0-DPA ovules were imaged by an S-3400 N SEM (Hitachi, Japan). From the similar region of each SEM image, an area of 250 × 250 μm^2^ was selected for fiber initial counting.

### Field experiment

Homozygous T_2_ generation lines of *35S::CKXRNAi* were planted in 2010 in an experimental farm at Southwest University (Chongqing, China) with 15 plants per row. Line CR-6 (T_4_) was selected as the best line of the moderately down-regulated *GhCKX* cotton.

To assess the agronomic performance of the line, plants were grown in field conditions for randomized comparative trial in 2012 with three replications. Each block was 20 m^2^ and contained 60 plants in four rows 1.0 m apart. The space between two neighboring plants in a row was 0.33 m. The plant height (from soil surface to top), fruit branch number, and square number were measured at 105 DAS. The boll number was counted at 130 DAS. After harvest, seed cotton was ginned. Fibers and seeds were weighed separately, and the number of seeds per boll, lint percentage (fiber weight/seed cotton weight), seed index (the weight of 100 seeds), lint index (the lint weight of 100 seeds), fiber yield and seed yield were determined. Fibers (3 × ~15 g) from each line were sampled randomly and sent to the National Center for Evaluation of Fiber Quality (Anyang, China) for measuring their qualities.

## Results

### Down-regulation of *GhCKX* in cotton

**Fig. 1:**
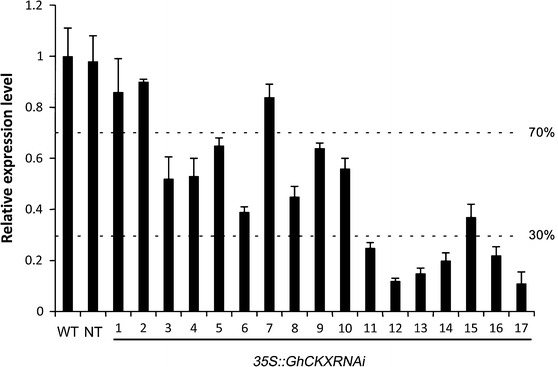
Relative expression levels of *GhCKX* in T_1_ transgenic cotton plants. RNAs were isolated from young stems of transgenic cotton plants and analyzed by real-time RT-PCR. *WT* wild type, *NT* nontransgenic plants segregated from selfed progenies of transgenic cottons. *Error bars* indicate SD of three replicates

To increase the cytokinin level in cotton by down-regulation of the expression of *GhCKX*, we generated transgenic *CaMV35S*::*GhCKXRNAi* (CR) cottons. 17 independent transgenic cottons were obtained. Based on *GhCKX* expression levels detected by real-time RT-PCR, transgenic cottons were divided into three groups: slightly suppressed (gene expression level was decreased by less than 30 %; CR-1, -2, and -7), moderately suppressed (decreased by 30–70 %; CR-3, -4, -5, -6, -8, -9, -10, and -15), and severely suppressed (over 70 %; CR-11, -12, -13, -14, -16, and -17) (Fig. 1). The severely suppressed *GhCKX* cottons showed a typical cytokinin overproduction phenotype, e.g. stunted shoots, shortened internode, smaller leaves and floral organs, and even sterility (CR-17, Fig. S1). In contrast, no developmental abnormality was observed in the slightly and moderately suppressed cottons.

### Down-regulation of *GhCKX*-enhanced endogenous cytokinins

To confirm that regulation of *GhCKX* changes the level of endogenous cytokinins in transgenic cottons, we determined the content of 13 different cytokinins in the leaves, stem shoots, flower buds, and ovules of two *GhCKX* moderately suppressed lines (CR-3 and -6) and one severely suppressed line (CR-13) by LC/MS (Tables 1 and S1). Total cytokinins in young leaves of transgenic cotton lines CR-3, -6 and -13 increased 13.6, 28.2, and 79.7 %, respectively, compared to that of the wild type. In mature leaves, the cytokinin level in transgenic lines increased 99.4, 106.7, and 194.5 %, respectively. For stem shoots, flower buds, and ovules, the averaged levels in CR-3 and CR-6 rose 39.8, 59.9, and 36.0 %, respectively, and the content in severely suppressed line CR-13 was rather high, increasing by 61.1, 315.6, and 139.9 %, respectively (Table 1). With the decline in gene expression, the content of cytokinins increased significantly, confirming that down-regulation of *GhCKX* elevated the level of endogenous cytokinins in cotton.

**Table 1:** Total content of cytokinins in transgenic lines and wild type (ng/g fresh weight)

Line	Cytokinins						
	Young leaf	Mature leaf	Stem shoot	Flower bud	Ovule	Total	Increase level (%)
WT	75.5	32.6	142.5	38.5	983.3	1,272.4	–
CR-3	85.8	65.0	190.3	53.0	1,138.2	1,532.3	20.4
CR-6	96.8	67.4	208.0	70.1	1,536.9	1,979.2	55.5
CR-13	135.7	96.0	229.6	160.0	2,359.1	2,980.4	134.2

Well-ground samples were extracted in cold 80 % (v/v) methanol. Cytokinins were purified by a Sep-Pak^®^ Plus tC18 cartridge (Waters, Ireland) and determined by HPLC–MS/MS systems (ABsciex, USA). The total values were the sum of cytokinin contents in young leaf, mature leaf, stem shoot, flower bud, and ovule, respectively. *WT* wild type; *CR*-*3*, *CR*-*6* and *CR*-*13*, *35S::GhCKXRNAi* transgenic plants

### Moderate down-regulation of *GhCKX* delayed leaf senescence

**Fig. 2:**
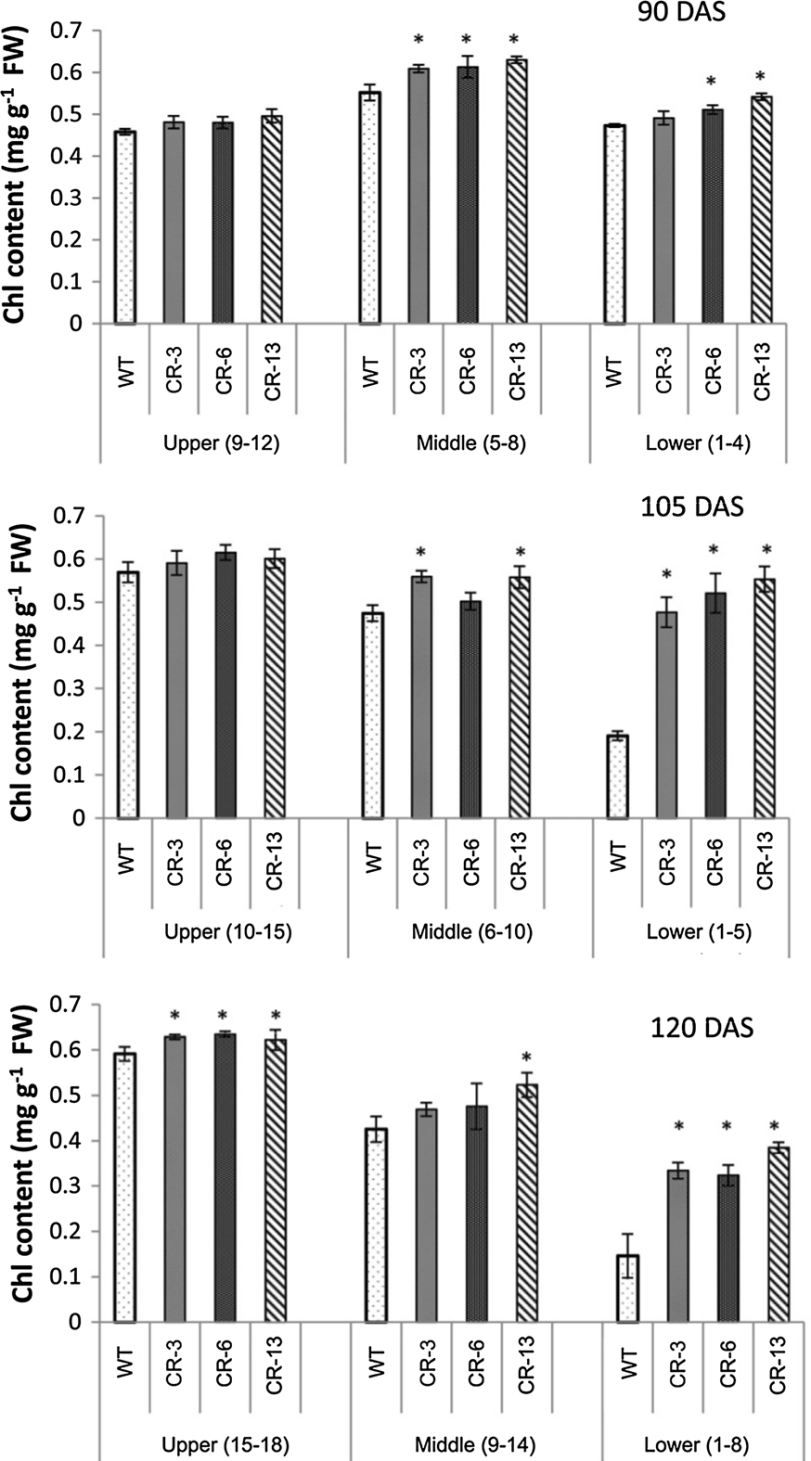
Chlorophyll contents of upper, middle, and lower leaves in 90-, 105- and 120-DAS plants. Results are presented as mean ± SD (*n* = 3). *Asterisks* (*) indicate a significant difference (*P* < 0.05). *FW* fresh weight

Chlorophyll degradation is a sign of leaf senescence (Kusaba et al. 2007). To investigate whether down-regulation of *GhCKX* in cotton delays the leaf senescence, we compared the chlorophyll content in the leaves of *GhCKX* moderately suppressed lines (CR-3 and -6), severely suppressed line (CR-13), and wild type at 90, 105, and 120 DAS in field conditions. For upper leaves, there was a steady increase in chlorophyll content in transgenic lines and the wild type from 90 DAS to 105 and 120 DAS (Fig. 2). The content in the three transgenic lines was higher than that in the wild type. For the middle leaves, along with aging, the content in 105- and 120-DAS leaves of transgenic lines and wild-type plants declined. However, the content in transgenic leaves was higher than the wild type. In the aged leaf, i.e. the 120-DAS lower leaves, the content decreased dramatically compared with the upper leaves (Fig. 2). Nevertheless, the content in the transgenic lines was significantly higher than the wild type at 105 and 120 DAS. We further calculated the number of abscised stem leaves per plant at 105, 120, and 140 DAS. During the time, leaf abscission in the cotton increased quickly (Fig. S2), even though the numbers of abscised leaves in the three transgenic lines was much lower than that in the wild type (Fig. S2). The assays of chlorophyll content and leaf abscission demonstrated that leaf senescence was delayed in the transgenic lines.

### Moderate down-regulation of *GhCKX* increased leaf photosynthesis and enhanced soluble sugar and protein in the boll

**Fig. 3:**
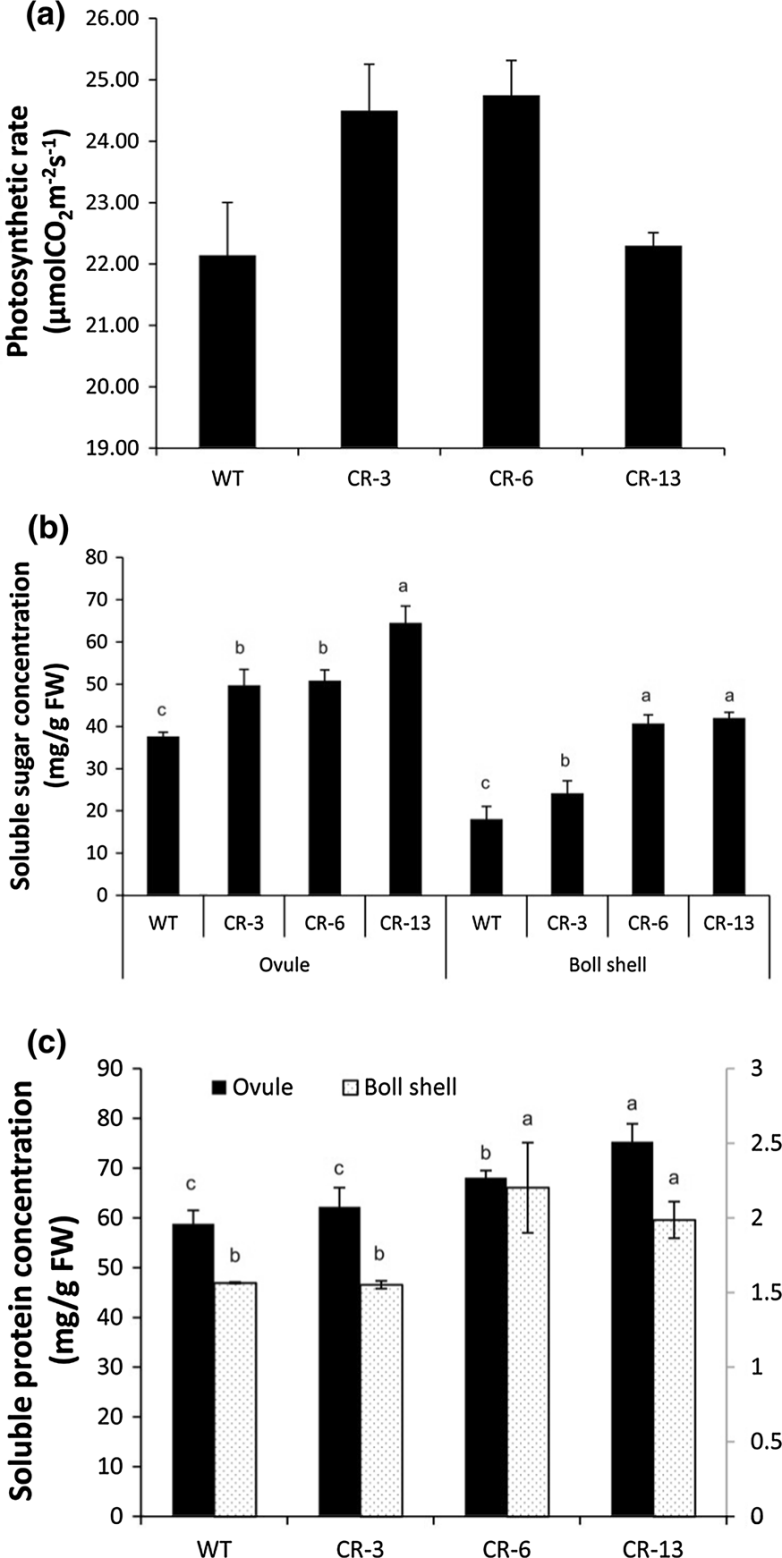
Comparison of photosynthetic rate in leaves, and soluble sugar and protein concentrations in bolls between transgenic lines and wild type. **a** Photosynthetic rate of the fourth leaf from the apex at 120 DAS. *Error bars* indicate SD of 10 randomly selected plants of each line. **b** Soluble sugar content of 35-DPA ovules and boll shells. **c** Soluble protein content of 35-DPA ovules and boll shells. *Error bars* indicate SD of data of three replicates for *each line*. For *each column*, means that are not followed by the *same letter* are significantly different according to Tukey’s range test at the 0.05 level

We measured photosynthesis of the fourth leaf from the apex at 120 DAS. The photosynthetic rate of CR-3 and CR-6 leaves was higher than the wild type, showing a 10.7 and 12.2 % increase, respectively. However, there was no such enhancement in the leaves of the severely suppressed line CR-13 (Fig. 3a). In addition, the concentrations of soluble sugar and protein in the boll shell and the ovule at 35 DPA (days post-anthesis) of CR-6 and CR-13 were significantly higher than those of the wild type (Fig. 3b, c).

### Moderate down-regulation of *GhCKX* increased both seed and lint yield

Based on the expression level of *GhCKX* (Fig. 1), coupled with phenotype observation in transgenic cottons (T_2_ generations), one slightly suppressed (the level decreased by less than 30 %) *GhCKX* line, CR-7, five moderately down-regulated lines, CR-3, -4, -5, -6, and -8 (decreased by 30–70 %), and two severely suppressed (decreased by over 70 %) lines, CR-11 and CR-13, were planted in field conditions to observe their agronomic performance. At the flower stage, the plant height of severely suppressed *GhCKX* lines CR-11 and -13 was 82.5 ± 13.7 cm and 68.7 ± 6.0 cm respectively, much lower than the control (100.5 ± 3.9 cm; Table S2). At the same time, the height of most of the moderately down-regulated lines showed a slight decreased, and there was not much change in the height between the slightly down-regulated line CR-7 and the wild type. Meanwhile, the moderately down-regulated lines bore more fruiting branches than the wild type. In the case of severely suppressed lines CR-11 and -13, the branch number noticeably decreased (Table S2), indicating that moderate down-regulation of *GhCKX* promoted the development of fruiting branches, but severe suppression of the gene hindered it. The square (flower bud) number at 105 DAS of most of the moderately down-regulated lines was higher, while the square number of the two severely suppressed lines was lower than that of the wild type. After harvest, we counted the boll number per plant and calculated the seed and lint fiber yields. The agronomic behavior of the slightly down-regulated line, CR-7, was very close to the wild type (Table S2). The moderately down-regulated cottons produced more bolls than the wild type. The averaged boll number of the five moderately down-regulated lines was 30.7 ± 1.7, 19.0 % more than the wild type. Moreover, bolls produced by these lines were bigger than the wild type, as indicated by the seed cotton weight per boll and seed index (the weight in grams of 100 seeds, Table S2). Consequently, seed cotton yield per plant of the lines increased significantly. In contrast, the number of bolls and the seed size in the severely suppressed cottons, CR-11 and CR-13, were lower and smaller than the wild type.

Interestingly, lint index (the weight in grams of lint in 100 seeds), a measure of the abundance of fiber on the seed, and the lint yield per plant increased in the moderately down-regulated lines, demonstrating that the enhancement of seeds was not at the cost of lint fibers; instead, both seed and lint yield were concurrently improved. However, boll number per plant, seed number and seed cotton per boll, and seed cotton yield and lint yield per plant of the severely suppressed lines CR-11 and CR-13 were dramatically lower than the wild type (Table S2), suggesting that over-dosage of endogenous cytokinins reduced both seed and lint yield. With regard to the quality of the fiber, we did not find notable alterations in fiber length, fiber strength, or micronaire in transgenic cottons (Table S3), suggesting that the increase of endogenous cytokinins in cotton had little influence on the qualities of transgenic fibers.

To further assess the performance of the moderately down-regulated *GhCKX* cotton in yield improvement, we selected CR-6 (T_4_) as the best line for field trials. As in T_2_ generation, yield components, including boll number per plant, seed cotton per boll, lint index and seed index, of the transgenic line were higher than that of the wild type. The seed yield and lint yield increased 15.4 and 20.0 %, respectively, as compared to the wild type (Table 2). The field trial results confirmed that moderate down-regulation of *GhCKX* enhanced both fiber and seed yield.

**Table 2:** Yield and fiber quality of transgenic line CR-6 and wild type in field trial

Line	Boll number	Seed cotton weight per boll (g)	Lint percentage (%)	Lint index (g)	Seed number per boll	Seed index (g)	Seed cotton yield (kg/plot)	Lint yield (kg/plot)	Seed yield (kg/plot)	Fiber length (mm)	Micronaire	Fiber strength (CN/tex)
WT	23.5 ± 1.3	4.6 ± 0.1	39.5 ± 0.2	6.9 ± 0.2	25.6 ± 0.3	10.6 ± 0.3	6.4 ± 0.3	2.5 ± 0.1	3.9 ± 0.2	30.8 ± 0.7	4.9 ± 0.1	31.2 ± 0.5
CR-6	25.6 ± 1.4	4.9 ± 0.1*	39.8 ± 0.5	7.4 ± 0.1*	26.0 ± 0.5	11.2 ± 0.1*	7.5 ± 0.4*	3.0 ± 0.2*	4.5 ± 0.2*	31.0 ± 0.2	5.0 ± 0.1	31.4 ± 0.7

Plants were grown in the farm at Southwest University (Chongqing, China) in a randomized comparative trial with three replications. The plot size was 25 m^2^. Results are presented as mean ± SD (*n* = 3)

Asterisk (*) indicates significant differences at *P* < 0.05 according to Student’s *t* test

## Discussion

Cotton fibers are derived from single cells of ovule epidermis (Stewart 1975) and fiber cell development is tightly influenced by seed (Ruan 2013). Phytohormones play important roles in the growth and development of seed and fiber cells of cotton (Beasley and Ting 1973; Basra and Saha 1999; Lee et al. 2007). Indole-3-acetic acid and gibberellins were able to promote fiber cell initiation and elongation (Chen et al. 1988, 1996; Zhang et al. 2011; Xiao et al. 2010). Ethylene plays a major role in promoting fiber elongation (Shi et al. 2006), whereas abscisic acid (ABA) inhibits fiber growth (Dhindsa et al. 1976; Gokani et al. 1998; Lee et al. 2007). Brassinosteroids (BRs) participate in both fiber initiation and elongation (Sun et al. 2005; Luo et al. 2007). The inhibitory effect of BRZ on fiber cells could be overcome by ethylene, indicating the cross-talk between BRs and ethylene (Shi et al. 2006). Cytokinins have an important role in shoot and seed development, but fiber elongation is inhibited by high concentrations of cytokinins in ovule culture (Beasley and Ting 1974). However, the effects of cytokinins on the yield of cotton fiber and seeds remained to be evaluated.

To elevate the endogenous level of cytokinins, we successfully generated *35S::GhCKXRNAi* cottons. A dose-dependent effect of the gene expression on phenotype alterations was observed. The slightly suppressed cottons did not show distinct morphological and agronomic changes relative to the wild type, while moderate suppression resulted in a delay in leaf senescence, increase in photosynthesis, more fruiting branches and bolls, bigger size of seed, and consequently higher yield of seed and fiber. Along with the decrease in *GhCKX* expression, the negative impact of cytokinin overproduction on plant growth and development became progressively more severe, and the yield of seed and fiber of the severely suppressed cottons dramatically declined. The cytokinin content assay was well consistent with the dosage change in gene expression. That is to say, along with the decline in gene expression, the endogenous level of cytokinins increased and the enhancement of endogenous cytokinins creates the alterations taking place in the transgenic cottons.

The increased cytokinin content caused an enhancement of seed yield in rice (Ashikari et al. 2005) and *Arabidopsis* (Bartrina et al. 2011). Our ovule culture results demonstrated that application of ZT (trans-Zeatin) at appropriate concentrations (5.0–8.0 μM) in media promoted seed development. However, a high concentration of the cytokinin (15 μM) inhibited seed development (Fig. S3). Results in transgenic *35S::GhCKXRNAi* cottons supported this observation. Ovules of the severely suppressed cotton contained high concentration of cytokinins (Tables 1 and S1) and the cotton plants produced less seeds with smaller size, whereas the seed number, seed size, and seed cotton yield were significantly enhanced in the moderately suppressed cottons (Table S2). These results indicated that a moderate enhancement of cytokinins promotes the development of seeds, but overdosage of them inhibits it.

In the ovule culture experiment, fiber elongation was inhibited in cytokinin-overproduction transgenic cottons (Yu et al. 2000). In our experiments, no distinct inhibition of fiber initiation was observed in moderately suppressed transgenic cottons (Fig. S4). Meanwhile, there was no distinct difference in fiber length between transgenic lines and the wild type (Table S3), implying that moderate overexpression of *GhCKXRNAi* has little negative effect on fiber development. Instead, lint index and lint yield of the moderately suppressed *GhCKX* lines were significantly enhanced. There are two explanations for this enhancement. First, the number of fibers per seed is limited by the surface area of the ovule epidermis. Thus, enhancement of cotton seed development could achieve cotton seed and fiber yield improvement (Ruan 2013). In our experiments, a proper increase of cytokinins promoted seed development and enlarge the seed size (Fig. S3). Bigger seed means larger area of seed coat that bears more fibers. More importantly, early leaf senescence could decrease the assimilation supply for boll and fiber development (Bauer et al. 2000; Peng and Krieg 1991), thus reducing lint yield and fiber properties (Wright 1999; Dong et al. 2006). The delay of leaf senescence in transgenic cottons leads to an increase in chlorophyll concentration and photosynthetic rate of the leaves. Our results are in agreement with previous findings that early-fruit removal significantly increased cytokinins in both main-stem leaves and xylem sap and delayed the main-stem leaf senescence characterized by increases in chlorophyll concentration and photosynthetic activity of leaves (Dong et al. 2009). The enhanced photosynthesis in the leaves of transgenic cotton plants and the increased soluble sugar and protein of the boll (Fig. 3) provide more carbohydrates for the development of seed and fiber. As a result, seed yield and fiber yield concurrently improve.

Although seed-specific expression of *ipt* can alleviate developmental abnormalities resulting from constitutive overexpression of the gene (Ma et al. 2002, 2008; Daskalova et al. 2007), the shortcoming of this strategy is that the advantages associated with increase in cytokinins, such as delay in leaf senescence, increase in photosynthesis of leaves, more branches, tolerance to abiotic and biotic stress (Choi et al. 2010; Jeon et al. 2010; Nishiyama et al. 2011; Peleg et al. 2011; Rivero et al., 2007), and resistance to pathogens (Choi et al. 2011), may not benefit the whole plant. In our previous experiments, we failed to generate transgenic *35S::ipt* cotton. In contrast, in this paper, using the strategy of suppressing cytokinin deactivation, we successfully obtained transgenic *35S::GhCKXRNAi* cottons in which endogenous cytokinin levels were globally and constitutively enhanced (Tables 1 and S1). In terms of manipulation of cytokinin levels in plants, *CKX* is more likely a softer regulator than *ipt*. Taken together, our data demonstrated that moderately enhancing endogenous cytokinins by suppressing *CKX* is a feasible and effective strategy for yield improvement, not only for cotton but also for other seed crops, such as canola, soybean, maize, and rice.

## Electronic supplementary material

Below is the link to the electronic supplementary material.

Supplementary material 1 (DOCX 823 kb)

## References

[CR1] Ashikari M, Sakakibara H, Lin S, Yamamoto T, Takashi T, Nishimura A, Angeles ER, Qian Q, Kitano H, Matsuoka M (2005). Cytokinin oxidase regulates rice grain production. Science.

[CR2] Bartrina I, Otto E, Strnad M, Werner T, Schmülling T (2011). Cytokinin regulates the activity of reproductive meristems, flower organ size, ovule formation, and thus seed yield in *Arabidopsis thaliana*. Plant Cell.

[CR3] Basra A, Saha S, Basra AS (1999). Growth regulation of cotton fibers. Cotton fibers: developmental biology, quality improvement, and textile processing.

[CR4] Bauer PJ, Frederick JR, Bradow JM, Sadler EJ, Evans DE (2000). Canopy photosynthesis and fiber properties of normal- and late-planted cotton. Agron J.

[CR5] Beasley CA, Ting IP (1973). Effects of plant growth substances on in vitro fiber development from fertilized cotton ovules. Am J Bot.

[CR6] Beasley CA, Ting IP (1974). Effects of plant growth substances on in vitro fiber development from unfertilized cotton ovules. Am J Bot.

[CR7] Chen YN, Shen CY, Zhang ZL, Yan JQ (1988). Study of the fiber development of cotton ovules. Acta Biol Exp Sinica.

[CR8] Chen JG, Du XM, Zhao HY, Zhou X (1996). Fluctuation in levels of endogenous plant hormones in ovules of normal cotton during flowering and their relation to fiber development. J Plant Growth Regul.

[CR9] Choi J, Huh SU, Kojima M, Sakakibara H, Paek KH, Hwang I (2010). The cytokinin-activated transcription factor ARR2 promotes plant immunity via TGA3/NPR1-dependent salicylic acid signaling in Arabidopsis. Dev Cell.

[CR10] Choi J, Choi D, Lee S, Ryu CM, Hwang I (2011). Cytokinins and plant immunity: old foes or new friends?. Trends Plant Sci.

[CR11] Chory J, Reinecke D, Sim S, Washburn T, Brenner M (1994). A role for cytokinins in de-etiolation in *Arabidopsis* (*det* mutants have an altered response to cytokinins). Plant Physiol.

[CR12] Daskalova S, McCormac A, Scott N, Van Onckelen H, Elliott M (2007). Effect of seed-specific expression of the *ipt* gene on *Nicotiana tabacum* L. seed composition. Plant Growth Regul.

[CR13] Dhindsa RS, Beasley CA, Ting IP (1976). Effects of abscisic acid on in vitro growth of cotton fiber. Planta.

[CR14] Dong HZ, Li WJ, Tang W, Li ZH, Zhang DM, Niu YH (2006). Yield, quality and leaf senescence of cotton grown at varying planting dates and plant densities in the Yellow river valley of China. Field Crops Res.

[CR15] Dong HZ, Niu YH, Kong XQ, Luo Z (2009). Effects of early-fruit removal on endogenous cytokinins and abscisic acid in relation to leaf senescence in cotton. Plant Growth Regul.

[CR16] Gan S, Amasino RM (1995). Inhibition of leaf senescence by autoregulated production of cytokinin. Science.

[CR17] Geng S, Ma M, Ye HC, Liu BY, Li GF, Chong K (2001). Effects of *ipt* gene expression on the physiological and chemical characteristics of *Artemisia annua* L. Plant Sci.

[CR18] Geng S, Ma M, Ye HC, Li GF (2002). Anther-specific expression of *ipt* gene in transgenic tobacco and its effect on plant development. Transgenic Res.

[CR19] Gokani SJ, Kumar R, Thaker VS (1998). Potential role of abscisic acid in cotton fiber and ovule development. J Plant Growth Regul.

[CR20] Guo J, Hu X, Duan R (2005). Interactive effects of cytokinins, light, and sucrose on the phenotypes and the syntheses of anthocyanins and lignins in cytokinin overproducing transgenic *Arabidopsis*. J Plant Growth Regul.

[CR21] Jeon J, Kim NY, Kim S, Kang NY, Novák O, Ku SJ, Cho C, Lee DJ, Lee EJ, Strnad M (2010). A subset of cytokinin two-component signaling system plays a role in cold temperature stress response in *Arabidopsis*. J Biol Chem.

[CR22] Jones RJ, Schreiber BMN (1997). Role and function of cytokinin oxidase in plants. Plant Growth Regul.

[CR23] Klee HJ, Horsch RB, Rogers SG (1987). *Agrobacterium*-mediated plant transformation and its further applications to plant biology. Annu Rev Plant Physiol.

[CR24] Kowalska M, Galuszka P, Frébortová J, Šebela M, Béres T, Hluska T, Šmehilová M, Bilyeu KD, Frébort I (2010). Vacuolar and cytosolic cytokinin dehydrogenases of *Arabidopsis thaliana*: heterologous expression, purification and properties. Phytochem.

[CR25] Kuppu S, Mishra N, Hu R, Sun L, Zhu X, Shen G, Blumwald E, Payton P, Zhang H (2013). Water-deficit inducible expression of a cytokinin biosynthetic gene *IPT* improves drought tolerance in cotton. PLoS ONE.

[CR26] Kusaba M, Ito H, Morita R, Iida S, Sato Y, Fujimoto M, Kawasaki S, Tanaka R, Hirochika H, Nishimura M, Tanaka A (2007). Rice NON-YELLOW COLORING1 is involved in light-harvesting complex II and grana degradation during leaf senescence. Plant Cell.

[CR27] Lee JJ, Woodward AW, Chen ZJ (2007). Gene expression changes and early events in cotton fibre development. Ann Bot.

[CR28] Li Y, Duan H, Wu Y, McAvoy R, Pei Y, Zhao DG, Wurst J, Li Q, Luo KM, Liang GH, Skinner DZ (2004). Transgenics of plant hormones and their potential application in horticultural crops. Genetically modified crops, their development, uses, and risks.

[CR29] Luo M, Xiao YH, Li XB, Lu XF, Deng W, Li DM, Hou L, Hu MY, Li Y, Pei Y (2007). GhDET2, a steroid 5α-reductase, plays an important role in cotton fiber cell initiation and elongation. Plant J.

[CR30] Ma QH, Lin ZB, Fu DZ (2002). Increased seed cytokinin levels in transgenic tobacco influence embryo and seedling development. Funct Plant Biol.

[CR31] Ma QH, Wang XM, Wang ZM (2008). Expression of isopentenyl transferase gene controlled by seed-specific lectin promoter in transgenic tobacco influences seed development. J Plant Growth Regul.

[CR32] Mähönen AP, Bishopp A, Higuchi M, Nieminen KM, Kinoshita K, Törmäkangas K, Ikeda Y, Oka A, Kakimoto T, Helariutta Y (2006). Cytokinin signaling and its inhibitor AHP6 regulate cell fate during vascular development. Science.

[CR33] McKenzie MJ, Jameson PE, Poulter RT (1994). Cloning an *ipt* gene from *Agrobacterium tumefaciens*: characterisation of cytokinins in derivative transgenic plant tissue. Plant Growth Regul.

[CR34] Medford JI, Horgan R, El-Sawi Z, Klee HJ (1989). Alterations of endogenous cytokinins in transgenic plants using a chimeric isopentenyl transferase gene. Plant Cell.

[CR35] Miller PA, Rawlings JQ (1967). Selection for increased lint yield and correlated responses in upland cotton, *Gossypium hirsutum* L. Crop Sci.

[CR36] Mok DW, Mok MC (2001). Cytokinin metabolism and action. Annu Rev Plant Physiol Plant Mol Biol.

[CR37] Nishiyama R, Watanabe Y, Fujita Y, Le DT, Kojima M, Werner T, Vankova R, Yamaguchi-Shinozaki K, Shinozaki K, Kakimoto T, Sakakibara H, Schmülling T, Tran LS (2011). Analysis of cytokinin mutants and regulation of cytokinin metabolic genes reveals important regulatory roles of cytokinins in drought, salt and abscisic acid responses, and abscisic acid biosynthesis. Plant Cell.

[CR38] Peleg Z, Reguera M, Tumimbang E, Walia H, Blumwald E (2011). Cytokinin-mediated source/sink modifications improve drought tolerance and increase grain yield in rice under water-stress. Plant Biotechnol J.

[CR39] Peng S, Krieg DR (1991). Single leaf and canopy photosynthesis response to plant age in cotton. Agron J.

[CR40] Reguera M, Peleg Z, Abdel-Tawab YM, Tumimbang EB, Delatorre CA, Blumwald E (2013). Stress-induced cytokinin synthesis increases drought tolerance through the coordinated regulation of carbon and nitrogen assimilation in rice. Plant Physiol.

[CR41] Rivero RM, Kojima M, Gepstein A, Sakakibara H, Mittler R, Gepstein S, Blumwald E (2007). Delayed leaf senescence induces extreme drought tolerance in a flowering plant. Proc Natl Acad Sci USA.

[CR42] Roitsch T, Ehneß R (2000). Regulation of source/sink relations by cytokinins. Plant Growth Regul.

[CR43] Ruan Y (2013). Boosting seed development as a new strategy to increase cotton fiber yield and quality. J Integr Plant Biol.

[CR44] Rupp HM, Frank M, Werner T, Strnad M, Schmülling T (1999). Increased steady state mRNA levels of the *STM* and *KNAT1* homeobox genes in cytokinin overproducing *Arabidopsis thaliana* indicate a role for cytokinins in the shoot apical meristem. Plant J.

[CR45] Sawan ZM, Mohamed AA, Sakr RA, Tarrad AM (2000). Effect of kinetin concentration and methods of application on seed germination, yield components, yield and fiber properties of the Egyptian cotton (*Gossypium barbadense*). Environ Exp Bot.

[CR46] Schmülling T, Werner T, Riefler M, Krupková E, Bartrina y Manns I (2003). Structure and function of cytokinin oxidase/dehydrogenase genes of maize, rice, *Arabidopsis* and other species. J Plant Res.

[CR47] Séguéla M, Briat JF, Vert G, Curie C (2008). Cytokinins negatively regulate the root iron uptake machinery in Arabidopsis through a growth-dependent pathway. Plant J.

[CR48] Shi YH, Zhu SW, Mao XZ, Feng JX, Qin YM, Zhang L, Cheng J, Wei LP, Wang ZY, Zhu YX (2006). Transcriptome profiling, molecular biological, and physiological studies reveal a major role for ethylene in cotton fiber cell elongation. Plant Cell.

[CR49] Siemens J, Keller I, Sarx J, Kunz S, Schuller A, Nagel W, Schmülling T, Parniske M, Ludwig-Müller J (2006). Transcriptome analysis of Arabidopsis clubroots indicate a key role for cytokinins in disease development. Mol Plant Microbe Interact.

[CR50] Smart CM, Scofield SR, Bevan MW, Dyer TA (1991). Delayed leaf senescence in tobacco plants transformed with tmr, a gene for cytokinin production in *Agrobacterium*. Plant Cell.

[CR51] Smigocki AC (1991). Cytokinin content and tissue distribution in plants transformed by a reconstructed isopentenyl transferase gene. Plant Mol Biol.

[CR52] Smigocki AC, Owens LD (1988). Cytokinin gene fused with a strong promoter enhances shoot organogenesis and zeatin levels in transformed plant cells. Proc Natl Acad Sci USA.

[CR53] Smigocki AC, Owens LD (1989). Cytokinin-to-auxin ratios and morphology of shoots and tissues transformed by a chimeric isopentenyl transferase gene. Plant Physiol.

[CR54] Stewart JM (1975). Fiber initiation on the cotton ovule (*Gossypium**hirsutum*). Am J Bot.

[CR55] Sun Y, Veerabomma S, Abdel-Mageed HA, Fokar M, Asami T, Yoshida S, Allen RD (2005). Brassinosteroid regulates fiber development on cultured cotton ovules. Plant Cell Physiol.

[CR56] Sunilkumar G, Campbell LM, Puckhaber L, Stipanovic RD, Rathore KS (2006). Engineering cottonseed for use in human nutrition by tissue-specific reduction of toxic gossypol. Proc Natl Acad Sci USA.

[CR57] Synková H, Van Loven K, Pospíšilová J, Valcke R (1999). Photosynthesis of transgenic P*ssu*-*ipt* tobacco. J Plant Physiol.

[CR58] van der Graaff EE, Auer CA, Hooykaas PJ (2001). Altered development of *Arabidopsis thaliana* carrying the *Agrobacterium tumefaciens ipt* gene is partially due to ethylene effects. Plant Growth Regul.

[CR59] Wang J, Letham DS, Cornish E, Stevenson KR (1997). Studies of cytokinin action and metabolism using tobacco plants expressing either the *ipt* or the *GUS* gene controlled by a chalcone synthase promoter. I. Developmental features of the transgenic plants. Aust J Plant Physiol.

[CR60] Werner T, Schmülling T (2009). Cytokinin action in plant development. Curr Opin Plant Biol.

[CR61] Werner T, Motyka V, Strnad M, Schmülling T (2001). Regulation of plant growth by cytokinin. Proc Natl Acad Sci USA.

[CR62] Werner T, Motyka V, Laucou V, Smets R, Van Onckelen H, Schmülling T (2003). Cytokinin-deficient transgenic Arabidopsis plants show multiple developmental alterations indicating opposite functions of cytokinins in the regulation of shoot and root meristem activity. Plant Cell.

[CR63] Wilkins TA, Rajasekaran K, Anderson DM (2000). Cotton biotechnology. Crit Rev Plant Sci.

[CR64] Wolters H, Jürgens G (2009). Survival of the flexible: hormonal growth control and adaptation in plant development. Nat Rev Genet.

[CR65] Wright PR (1999). Premature senescence of cotton (*Gossypium**hirsutum* L.)—predominantly a potassium disorder caused by an imbalance of source and sink. Plant Soil.

[CR66] Xiao YH, Li DM, Yin MH, Li XB, Zhang M, Wang YJ, Dong J, Zhao J, Luo M, Luo XY, Hou L, Hu L, Pei Y (2010). Gibberellin 20-oxidase promotes initiation and elongation of cotton fibers by regulating gibberellin synthesis. J Plant Physiol.

[CR67] Yu XH, Zhu YQ, Lu S, Zhang TZ, Chen XY, Xu ZH (2000). A comparative analysis of a *fuzzless*-*lintless* mutant of *Gossypium hirsutum* L. cv. Xu-142. Sci China.

[CR68] Zeng QW, Qin S, Song SQ, Zhang M, Xiao YH, Luo M, Hou L, Pei Y (2012). Molecular cloning and characterization of a cytokinin dehydrogenase gene from upland cotton (*Gossypium hirsutum* L.). Plant Mol Biol Rep.

[CR69] Zhang JF, Lu Y, Cantrell RG, Hughs E (2005). Molecular marker diversity and field performance in commercial cotton cultivars evaluated in the Southwestern USA. Crop Sci.

[CR70] Zhang M, Zheng XL, Song SQ, Zeng QW, Hou L, Li DM, Zhao J, Wei Y, Li XB, Luo M, Xiao YH, Luo XY, Zhang JF, Xiang CB, Pei Y (2011). Spatiotemporal manipulation of auxin biosynthesis in cotton ovule epidermal cells enhances fiber yield and quality. Nat Biotechnol.

