# Supplementary material for: Moderately enhancing cytokinin level by down-regulation of GhCKX expression in cotton concurrently increases fiber and seed yield
Source: Mol Breed. 2015 Jan 29;35(2):60. doi: 10.1007/s11032-015-0232-6 (PMC4309883; doi:10.1007/s11032-015-0232-6)
Supplement: Supplementary file 1 — Supplementary material 1 (DOCX 823 kb) [file 11032_2015_232_MOESM1_ESM.docx]

**Supporting information**

**Table S1** Contents of 13 different cytokinins in leaves, stem shoots, flower buds, and ovules, respectively

**Table S2** Comparison of plant growth and yield components in transgenic lines and wild type

**Table S3** Fiber quality of transgenic cotton and wild type

**Table S4** Acquisition parameters of cytokinins and the corresponding deuterated internal standards

**Figure S1** *GhCKX* severely-suppressed cotton showed stunted shoots, shorten internodes, smaller leaves and floral organs

**Figure S2** Leaf senescence and leaf abscission in transgenic cottons and wild type

**Figure S3** Effect of ZT on ovule development *in vitro*

**Figure S4** Fiber initial densities on the surface of 0 DPA ovules

**Table S1** Contents of 13 different cytokinins in leaves, stem shoots, flower buds, and ovules, respectively (ng/g FW)

| Tissue | Line | IP | ZT | ZR | IPR | IP9G | ZOG | Z9G | DHZ | DZOG | DZ9G | ZROG | DZR | DZROG |
| --- | --- | --- | --- | --- | --- | --- | --- | --- | --- | --- | --- | --- | --- | --- |
| Young leaf | WT | 2.1±0.0 | 7.1±1.0 | 27.9±0.2 | 0.3±0.0 | 0.2±0.0 | 2.7±0.8 | 7.5±0.1 | 2.0±0.1 | 22.8±0.7 | 0.2 ±0.0 | 2.1±0.5 | 0.2±0.1 | 0.4±0.0 |
|  | CR-3 | 2.0±0.1 | 7.4±1.1 | 37.8±2.7 | 0.4±0.1 | 0.2±0.0 | 2.9±0.2 | 4.4±0.1 | 2.5±0.0 | 25.1±0.6 | 0.3 ±0.1 | 2.4±0.6 | 0.3±0.1 | 0.1±0.0 |
|  | CR-6 | 2.5±0.1 | 9.2±0.7 | 37.9±3.4 | 0.6±0.0 | 0.3±0.1 | 4.4±0.4 | 2.0±0.1 | 2.4±0.4 | 32.8±1.8 | 0.8 ±0.1 | 2.8±0.1 | 0.8±0.1 | 0.3±0.0 |
|  | CR-13 | 3.2±0.1 | 12.3±1.3 | 55.1±3.1 | 0.7±0.1 | 0.3±0.1 | 4.5±0.8 | 2.5±0.1 | 4.4±0.5 | 48.6±1.0 | 0.6±0.1 | 2.7±0.2 | 0.6±0.0 | 0.2±0.0 |
| Mature leaf | WT | 1.3±0.0 | 2.7±0.3 | 1.8±0.0 | 1.4±0.1 | 0.2±0.0 | 8.6±0.5 | 0.0±0.0 | 0.4±0.1 | 10.7±1.6 | 0.1±0.0 | 5.2±1.2 | 0.1±0.0 | 0.1±0.0 |
|  | CR-3 | 1.6±0.0 | 3.9±0.2 | 2.5±0.6 | 1.3±0.1 | 0.1±0.0 | 10.3±0.9 | 0.1±0.0 | 1.6±0.1 | 26.6±1.0 | 0.2±0.0 | 16.5±0.1 | 0.2±0.1 | 0.1±0.0 |
|  | CR-6 | 1.7±0.2 | 4.2±0.9 | 3.6±0.4 | 1.4±0.0 | 0.3±0.0 | 9.9±1.5 | 0.1±0.0 | 2.8±0.3 | 27.1±2.5 | 0.2±0.0 | 15.8±2.3 | 0.2±0.0 | 0.1±0.0 |
|  | CR-13 | 2.8±0.1 | 9.9±0.4 | 5.0±1.4 | 2.2±0.3 | 0.5±0.0 | 15.0±0.9 | 5.6±0.3 | 5.4±0.2 | 32.2±0.7 | 0.3±0.1 | 16.7±1.3 | 0.3±0.0 | 0.1±0.0 |
| Stem  shoot | WT | 2.3±0.1 | 1.6±0.5 | 0.5±0.0 | 9.2±0.8 | 116.3±5.8 | 3.5±0.2 | 0.4±0.0 | 0.0±0.0 | 2.1±0.2 | 0.1±0.0 | 6.2±0.2 | 0.3±0.1 | 0.0±0.0 |
|  | CR-3 | 3.0±0.1 | 1.7±0.2 | 0.9±0.0 | 23.2±0. 9 | 150.1±7.9 | 2.5±0.1 | 0.2±0.0 | 0.1±0.1 | 0.5±0.1 | 0.1±0.0 | 7.3±0.2 | 0.7±0.2 | 0.0±0.0 |
|  | CR-6 | 4.6±0.2 | 2.0±0.1 | 1.0±0.1 | 26.7±0.8 | 160.3±5.6 | 2.3±0.5 | 0.3±0.0 | 0.1±0.0 | 1.1±0.3 | 0.2±0.0 | 9.1±0.7 | 0.3±0.2 | 0.0±0.0 |
|  | CR-13 | 15.4±0.2 | 4.6±0.1 | 1.1±0.1 | 27.0±1.0 | 162.3±11.1 | 3.3±0.1 | 0.4±0.0 | 0.1±0.0 | 2.5±0.2 | 1.1±0.1 | 11.1±0.6 | 0.7±0.2 | 0.0±0.0 |
| Flower  bud | WT | 7.4±0.3 | 3.9±0.9 | 0.9±0.1 | 2.4±1.0 | 4.6±0.6 | 5.6±0.8 | 0.2±0.0 | 1.1±0.0 | 1.3±0.7 | 0.0±0.0 | 10.0±0.6 | 0.5±0.1 | 0.6±0.0 |
|  | CR-3 | 9.8±0.1 | 4.2±0.6 | 1.1±0.3 | 4.0±0.1 | 6.9±0.3 | 5.3±0.3 | 0.3±0.0 | 1.6±0.2 | 1.3±0.5 | 0.0±0.0 | 17.7±0.2 | 0.4±0.2 | 0.4±0.1 |
|  | CR-6 | 9.8±0.7 | 9.0±0.1 | 1.8±0.2 | 7.9±0.9 | 5.1±0.1 | 3.3±0.2 | 0.3±0.0 | 1.6±0.1 | 3.0±0.9 | 0.2±0.0 | 26.5±2.2 | 1.3±0.1 | 0.3±0.0 |
|  | CR-13 | 16.6±0.3 | 12.7±0.5 | 4.4±0.5 | 15.4±1.4 | 25.5±0.5 | 6.1±0.2 | 0.5±0.0 | 2.6±0.1 | 4.8±1.2 | 0.0±0.0 | 69.9±11.2 | 0.6±0.0 | 0.9±0.1 |
| Ovule | WT | 3.3±0.1 | 2.1±0.2 | 3.8±0.1 | 1.2±0.5 | 951.1±25.8 | 1.3±0.1 | 3.9±0.2 | 2.3±0.3 | 0.1±0.0 | 0.9±0.1 | 9.1±0.4 | 0.7±0.1 | 3.5±0.1 |
|  | CR-3 | 6.6±0.1 | 3.5±0.1 | 4.9±0.0 | 1.5±0.2 | 1099.9±18.7 | 0.6±0.1 | 3.9±1.1 | 2.0±0.1 | 0.2±0.1 | 0.2±0.1 | 11.0±0.5 | 0.8±0.1 | 3.1±0.1 |
|  | CR-6 | 8.0±0.1 | 7.6±0.1 | 6.0±0.5 | 2.3±0.3 | 1481.9±60.7 | 3.5±0.7 | 6.6±0.4 | 3.1±0.2 | 0.3±0.1 | 0.2±0.1 | 10.4±0.2 | 1.2±0.2 | 5.8±0.2 |
|  | CR-13 | 14.9±0.1 | 7.6±0.0 | 11.6±0.5 | 4.0±0.4 | 2278.1±68.6 | 1.8±0.7 | 15.8±0.6 | 3.7±0.1 | 0.2±0.1 | 0.9±0.2 | 15.2±0.3 | 1.2±0.4 | 4.1±0.3 |

IP, isopentenyl adenine; ZT, trans-zeatin; ZR, trans-zeatin riboside; IPR, isopentenyl adenosine; IP9G, isopentenyl adenine 9-glucoside; ZOG, trans-zeatin O-glucoside; Z9G, trans-zeatin 9-glucoside; DHZ, dihydro-zeatin; DZOG, dihydro-zeatin O-glucoside; DZ9G, dihydro-zeatin 9-glucoside; ZROG, trans-zeatin riboside O-glucoside; DZR, dihydro-zeatin riboside; DZROG, dihydro-zeatin riboside O-glucoside.

**Table S2** Comparison of plant growth and yield components in transgenic lines and wild type

| Name | Plant  height(cm) | Fruiting branch number | Square number | Boll number | Seed cotton weight per boll (g) | Seed number per boll | Seed cotton yield per plant (g) | Lint (%) | Lint index (g) | Lint yield per plant (g) | Seed index (g) | Seed yield per plant (g) |
| --- | --- | --- | --- | --- | --- | --- | --- | --- | --- | --- | --- | --- |
| WT | 100.5±3.9 | 13.8±1.4 | 35.1±1.9 | 25.8±1.0 | 3.4±0.1 | 21.4±1.9 | 87.7 | 33.6±0.7 | 5.3±0.1 | 29.5 | 10.6±0.1 | 58.2 |
| CR-3 | 93.6±2.3 | 14.8±0.5 | 38.0±3.1 | 30.0±2.1 | 3.7±0.1 | 22.0±1.7 | 111.0 | 32.8±0.6 | 5.5±0.1 | 36.4 | 11.3±0.3 | 74.6 |
| CR-4 | 95.7±3.8 | 14.7±0.5 | 34.7±2.5 | 28.0±2.9 | 3.8±0.2 | 22.5±1.7 | 106.4 | 33.1±0.7 | 5.6±0.1 | 35.2 | 11.3±0.4 | 71.2 |
| CR-5 | 88.5±5.0 | 14.2±1.1 | 36.8±4.1 | 31.8±1.1 | 3.7±0.2 | 20.9±1.7 | 117.7 | 32.8±0.6 | 5.8±0.1 | 38.6 | 11.9±0.2 | 79.1 |
| CR-6 | 91.0±3.6 | 14.3±0.6 | 41.3±1.7 | 32.3±1.4 | 3.8±0.2 | 22.0±1.9 | 122.7 | 32.4±0.9 | 5.6±0.0 | 39.8 | 11.7±0.3 | 82.9 |
| CR-7 | 101.7±2.9 | 15.3±1.3 | 31.5±5.3 | 25.5±2.8 | 3.3±0.1 | 19.6±0.7 | 84.2 | 32.1±0.6 | 5.3±0.1 | 27.0 | 11.4±0.1 | 57.2 |
| CR-8 | 93.0±5.4 | 15.2±2.1 | 39.3±2.9 | 31.4±2.9 | 3.6±0.1 | 20.8±0.5 | 113.0 | 33.5±0.1 | 5.8±0.1 | 37.9 | 11.5±0.2 | 75.1 |
| CR-11 | 82.5±13.7 | 13.0±0.8 | 24.0±1.4 | 16.0±1.4 | 3.1±0.2 | 19.2±0.7 | 49.6 | 35.3±0.7 | 5.7±0.1 | 17.5 | 10.4±0.4 | 32.1 |
| CR-13 | 68.7±6.0 | 11.2±1.7 | 16.2±4.3 | 10.7±4.5 | 2.9±0.1 | 18.6±0.8 | 31.0 | 34.6±0.4 | 5.4±0.2 | 10.7 | 10.2±0.2 | 20.3 |

Seed cotton= seed + lint. Lint (%) = (lint weight/seed cotton weight)×100. Seed index, weight of 100 seeds; lint index, weight of lint from 100 seeds; yield per plant = boll number × seed cotton weight per boll. Data represents mean ± S.D. (n=15).

**Table S3** Fiber quality of transgenic cotton and wild type

| Name | Fiber length (mm) | Length uniformity  (%) | Micronaire | Elongation (%) | Fiber  strength  (CN/tex) |
| --- | --- | --- | --- | --- | --- |
| WT | 30.1±1.1 | 84.0±0.8 | 5.1±0.1 | 6.2±0.1 | 30.5±0.8 |
| CR-3 | 29.8±1.0 | 83.8±0.5 | 5.0±0.0 | 6.2±0.1 | 29.7±0.6 |
| CR-4 | 29.4±1.1 | 83.2±0.7 | 5.3±0.1 | 6.1±0.1 | 30.9±0.6 |
| CR-5 | 30.5±0.5 | 84.8±0.5 | 5.2±0.1 | 6.0±0.1 | 30.1±0.7 |
| CR-6 | 29.2±0.9 | 83.3±1.2 | 5.3±0.2 | 6.1±0.1 | 31.4±1.7 |
| CR-7 | 30.5±0.5 | 84.2±0.1 | 4.9±0.2 | 6.0±0.0 | 31.3±1.9 |
| CR-8 | 29.6±0.6 | 82.9±0.1 | 5.2±0.3 | 6.0±0.0 | 30.9±0.6 |
| CR-11 | 30.5±0.3 | 84.2±0.1 | 4.8±0.0 | 6.0±0.1 | 32.6±0.0 |
| CR-13 | 30.1±1.8 | 83.8±0.6 | 5.2±0.4 | 6.0±0.2 | 30.5±1.1 |

Values represent mean ± SD (n=3). CR, *35S::GhCKXRNAi* transgenic lines; WT, wild type. Fiber quality was analyzed at the National Center for Evaluation of Fiber Quality (Anyang, China).

**Table S4** Acquisition parameters of cytokinins and the corresponding deuterated internal standards

| Name | Q1 > Q3 (Da) | DP  (volts) | CE  (volts) | CXP  (volts) |
| --- | --- | --- | --- | --- |
| Z9G-D5 | 385.1 > 223.1 | -53.0 | -29.5 | -7.0 |
| Z9G | 380.0 > 218.0 | -45.0 | -24.0 | -11.0 |
| DZ9G-D3 | 385.1 > 223.1 | -53.0 | -29.5 | -7.0 |
| DZ9G | 382.0 > 220.0 | -53.0 | -21.0 | -11.5 |
| ZOG-D5 | 385.1 > 205.1 | -95.0 | -36.8 | -15.0 |
| ZOG | 380.0 > 200.0 | -95.0 | -33.0 | -9.8 |
| ZROG-D5 | 517.2 > 205.1 | -92.0 | -53.0 | -9.5 |
| ZROG | 512.2 > 200.0 | -92.0 | -51.0 | -9.5 |
| ZT-D5 | 223.0 > 133.0 | -82.0 | -35.1 | -9.1 |
| ZT | 218.0 > 133.0 | -82.0 | -22.0 | -9.8 |
| DZOG-D7 | 389.2 > 227.2 | -112.0 | -43.5 | -11.0 |
| DZOG | 382.2 > 220.0 | -112.0 | -40.0 | -11.3 |
| DZ-D3 | 223.0 > 133.0 | -87.0 | -40.5 | -4.1 |
| DHZ | 219.9 > 132.9 | -87.0 | -38.2 | -4.7 |
| DZROG-D7 | 521.2 > 389.3 | -95.0 | -36.0 | -8.0 |
| DZROG | 514.2 > 382.2 | -95.0 | -32.0 | -7.0 |
| ZR-D5 | 355.1 > 223.0 | -48.0 | -21.8 | -2.0 |
| ZR | 350.2 > 218.0 | -140.0 | -40.0 | -15.0 |
| DZR-D3 | 355.1 > 223.1 | -49.0 | -27.2 | -7.0 |
| DZR | 352.0 > 220.0 | -49.0 | -56.6 | -4.0 |
| IP9G-D6 | 370.1 > 208.1 | -47.2 | -24.0 | -10.0 |
| IP9G | 364.0 > 202.0 | -47.0 | -19.0 | -10.0 |
| IP-D6 | 208.0 > 133.0 | -75.0 | -30.2 | -10.7 |
| IP | 201.9 > 134.0 | -75.0 | -24.0 | -9.5 |
| IPR-D6 | 340.1 > 208.0 | -47.0 | -26.5 | -11.0 |
| IPR | 334.0 > 202.0 | -37.0 | -19.0 | -10.0 |

DP, declustering potential; CE, collision energy; CXP, collision cell exit potential.


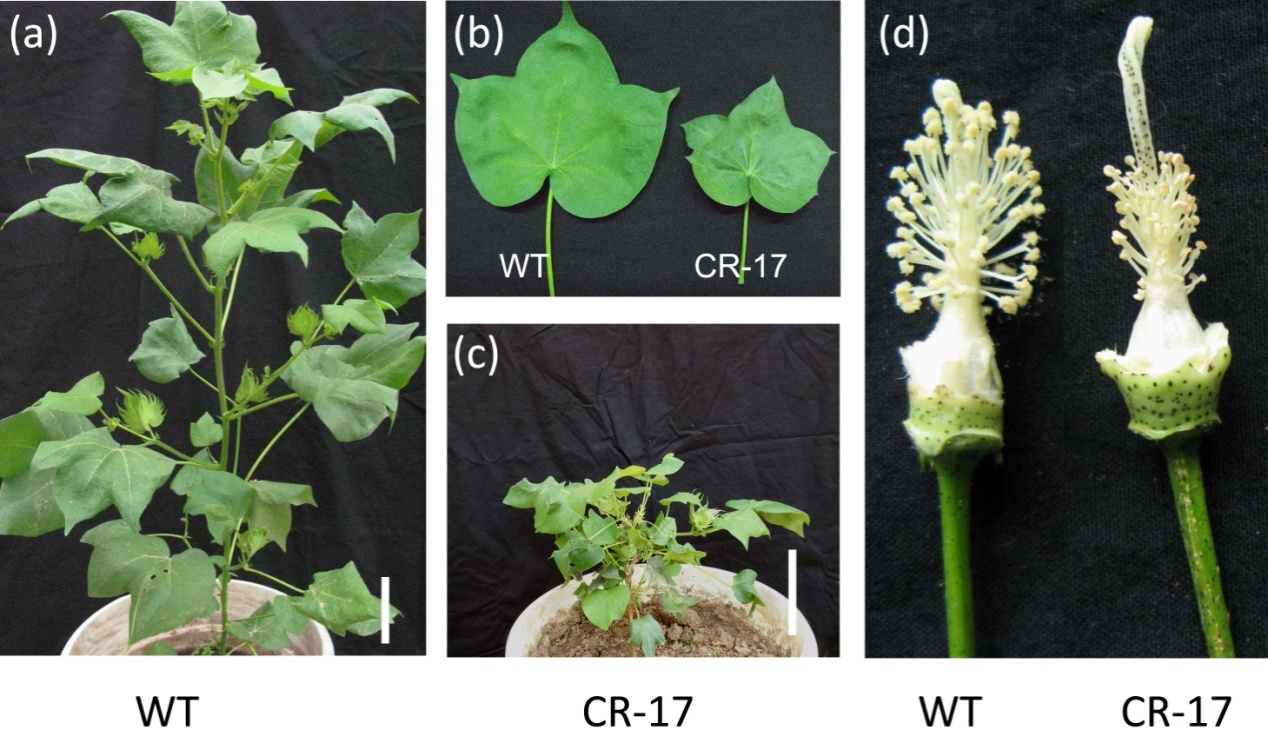


**Figure S1** *GhCKX* severely-suppressed cotton showed stunted shoots, shorten internodes, smaller leaves and floral organs. (a) Phenotypes of wild type (WT). Scale bar represents 10 cm. (b) Leaves derived from transgenic line and wild type. (c) Phenotypes of 35S::*GhCKXRNAi* transgenic plant No. 17. Scale bar represents 10 cm. (d) Stamens and pistils of transgenic cotton plant and wild type.


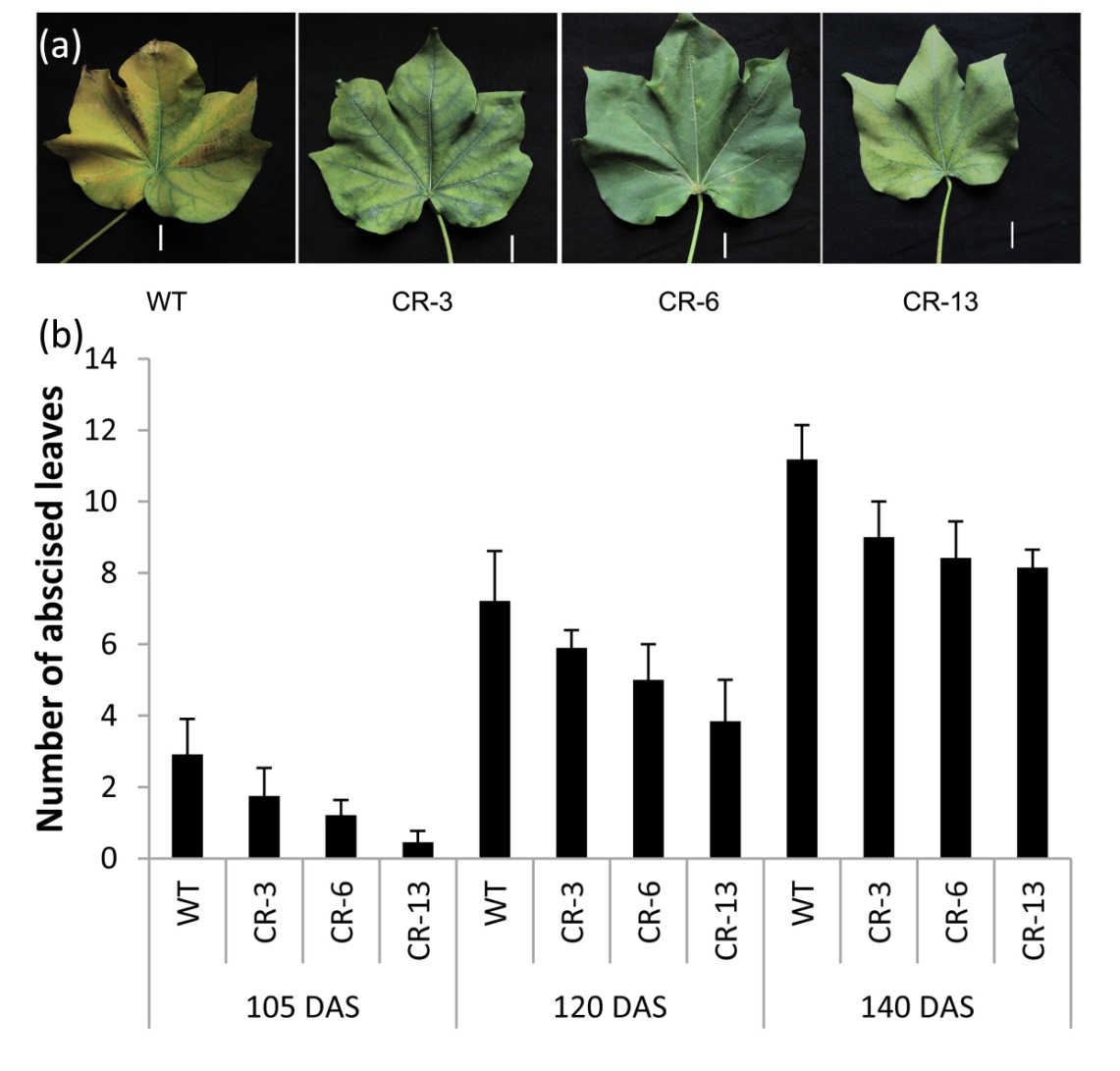


**Figure S2** Leaf senescence and leaf abscission in transgenic cottons and wild type. (a) Senescence was delayed in leaves (the tenth stem-leaf at 130 DAS) of transgenic plants. Scale bar represents 2 cm. (b) Leaf abscission number of 105-, 120- and 140- DAS stem-leaves in transgenic cotton and wild type. Error bars indicate S.D. of data of 15 randomly selected plants of each line.


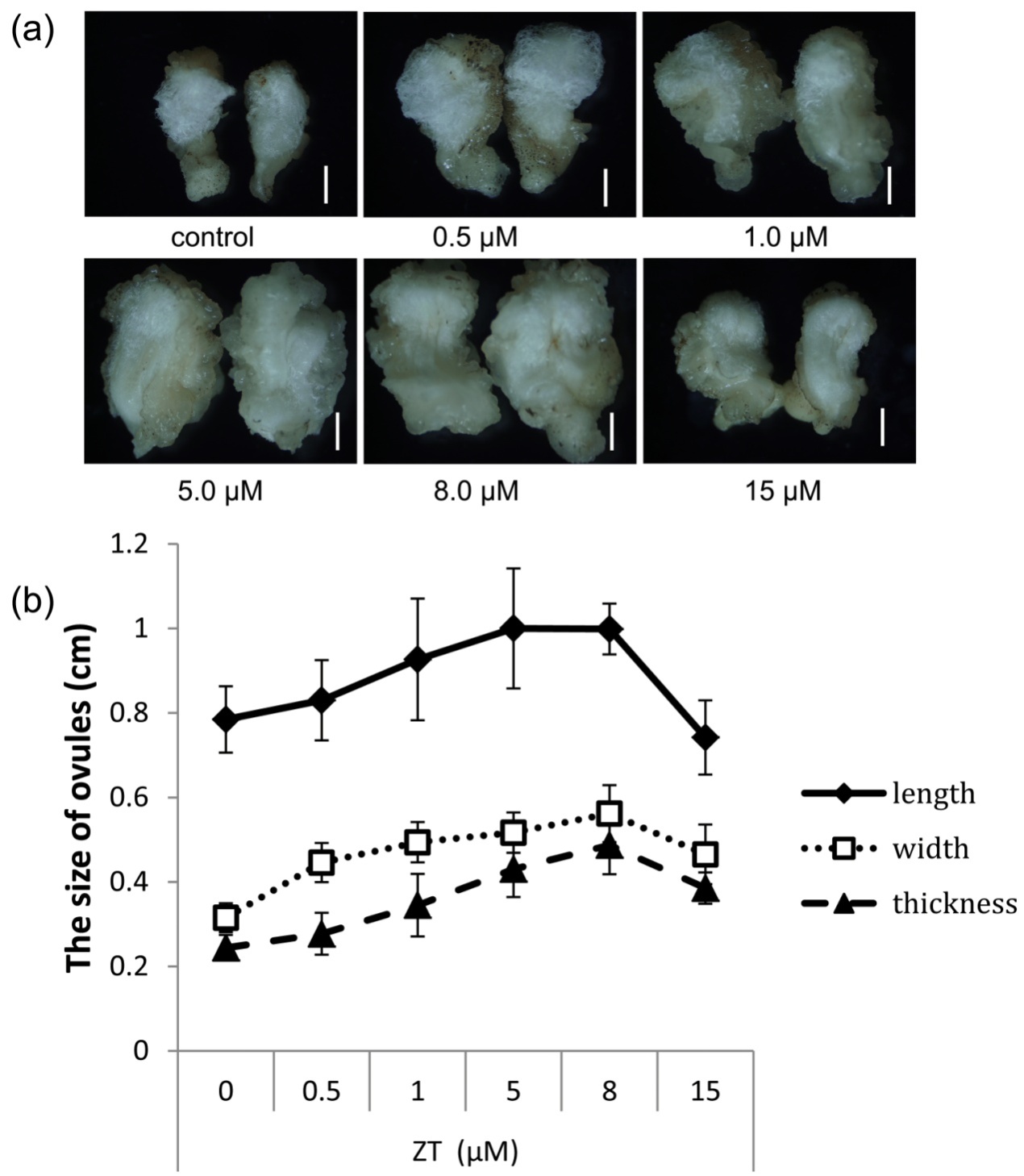


**Figure S3** Effect of ZT on ovule development *in vitro*. (a) Optical microscopic images of ovules. (b) Comparison of ovule length, width and thickness. The 0 DPA ovules at the middle position of bolls were incubated for two weeks in BT medium containing (0.5 μM GA_3_ and 5.0 μM IAA) plus with 0 μM, 0.5 μM, 1.0 μM, 5.0 μM, 8.0 μM and 15.0 μM ZT, respectively. Error bars indicate S.D. of data of 20 random ovules. Scale bars represent 2 mm.


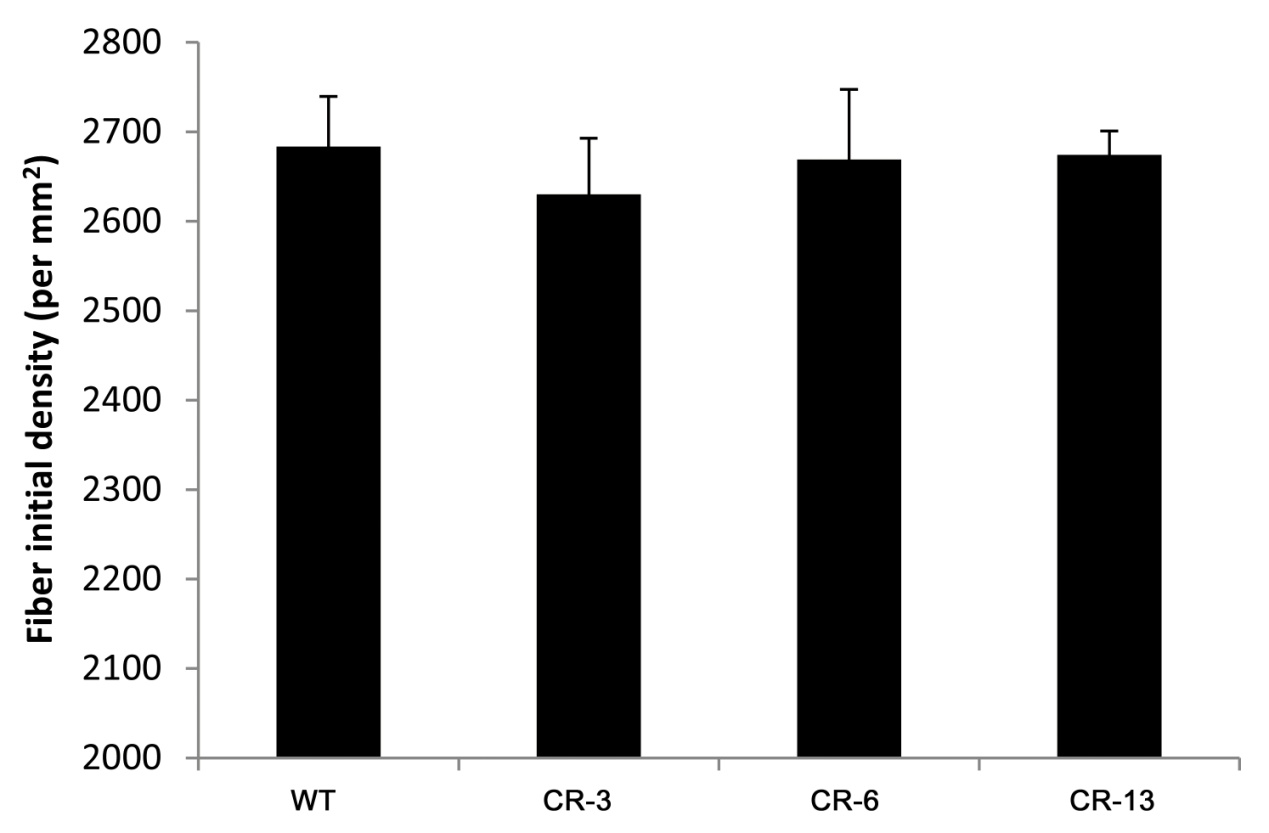


**Figure S4** Fiber initial densities on the surface of 0 DPA ovules. The results were based on the data of ten ovules from five bolls. Bars represent standard deviation.
